# Relative Association of Multi-Level Supportive Environments on Poor Health among Older Adults

**DOI:** 10.3390/ijerph14040387

**Published:** 2017-04-06

**Authors:** Nelda Mier, Marcia G. Ory, Samuel D. Towne, Matthew Lee Smith

**Affiliations:** 1 Department of Public Health Studies, Texas A&M School of Public Health, McAllen Campus, McAllen, TX 78503, USA; 2 Department of Health Promotion and Community Health Sciences, Texas A&M School of Public Health, College Station, TX 77843, USA; mory@sph.tamhsc.edu (M.G.O.); towne@sph.tamhsc.edu (S.D.T.); health@uga.edu (M.L.S.); 3 Institute of Gerontology, Department of Health Promotion and Behavior, College of Public Health, The University of Georgia, Athens, GA 30602, USA

**Keywords:** older adult, health status, supportive environments, physical health, mental health, United States of America, aging

## Abstract

*Background*: The aging of the United States population poses significant challenges to American healthcare and informal caregiving systems. Additional research is needed to understand how health promotion programs and policies based on a socio-ecological perspective impact the health and well-being of older persons. The purpose of this study was to investigate personal characteristics and supportive environments associated with poor health among older individuals aged 65 and over. *Methods*: This study used a cross-sectional design and was guided by a conceptual framework developed by the authors to depict the relationship between personal characteristics and environments associated with poor health status. Environment types included in this study were family, home, financial, neighborhood, and healthcare. The sample was comprised of 1319 adults aged 65 years and older residing in Central Texas. From a random selection of households, participants were administered a mail-based survey created by a community collaborative effort. Descriptive statistics and three binary logistic regression models were fitted to examine associations with poor health status (i.e., physical, mental, and combined physical/mental). *Results*: Two personal characteristics (number of chronic conditions and educational level) were consistently related (*p* < 0.05) to health outcomes. Supportive family, home, financial, neighborhood, and health care environmental factors were shown to be related (*p* < 0.05) to various aspects of physical or mental health outcomes. *Conclusions*: Multidimensional factors including personal characteristics and protective environments are related to health status among older individuals. The unique roles of each environment can help inform public health interventions to create and enhance support for older adults to engage in healthful activities and improve their physical and mental health.

## 1. Introduction

The aging population in the United States has reached 43 million persons and is growing rapidly with the aging of the baby boomer cohort [1]. Currently, one out of seven Americans is aged 65 years and older, but by 2030 one out of five individuals in this country will be an older person [2]. The average life expectancy of this age group is 19.2 additional years [2], an increase of almost 3 years since 1980 [3]. This demographic shift represents a significant challenge for the healthcare system and public health public and private entities responsible for promoting a healthy aging process for this important segment of society.

An aging population that is able to maintain their health poses less complex and cumbersome challenges to their caregivers and an already overtaxed healthcare system. Since 2001 health care expenditures for the elderly increased three-fold totaling 414.3 billion dollars in 2011, with Medicare being the main payment source [4] Although 43% of noninstitutionalized elders report to have excellent or very good health [2], data indicate that the majority of older people suffer from at least one chronic condition, including hypertension (71%), dyslipidemia (60%), and arthritis (52%) [5]. Also, the prevalence rates of elders reporting functional limitations increased from 19.7% in 2000 to 26.1% in 2014 [6]. What’s more, nearly nine in ten older adults suffer from two or more chronic conditions [7].

These demographic and healthcare challenges faced by Americans require effective strategies that can reduce chronic disease and improve the physical and mental functionality of older individuals. Research shows that access to healthcare is not the main factor impacting wellbeing. Health status is also influenced by health-related behaviors, environmental factors, and social conditions [8,9,10]. According to Healthy People 2020, social conditions include economic opportunities, social interactions, resources and supports available at home, neighborhood and community, among other. In addition, Healthy People 2020 also highlights that these conditions explain to certain degree why some people are healthier than others and “why Americans more generally are not as healthy as they could be” [11]. 

Studies with aging populations show that personal and social and environmental factors remain strong health determinants across the life-course. Personal factors found to be associated with health in older adults are age [12,13], gender [12,13,14,15,16], race/ethnicity [12,13], education attainment [13,17,18,19]; and employment [12]. Research conducted in Europe, Asia, Africa, and Latin America with aging populations found several economic and social factors associated with broad health and independence outcomes, including: reduced retirement pensions [20]; community involvement and social support [21,22,23]; housing conditions [22,24,25]; income level [17,22,26,27,28]; forced retirement [29]; and feeling discriminated [24]. 

Multiple determinants of health must be addressed to improve overall population health [30]. Too often considerations of the aging population are neglected in discussions of health promotion strategies [31,32]. Thus applying a broad personal, social and environmental health determinants framework to aging research issues has the potential to make a significant contribution to the identification of health programs and policies that will benefit older persons in maintaining their health, independence, and function. 

**Figure 1:**
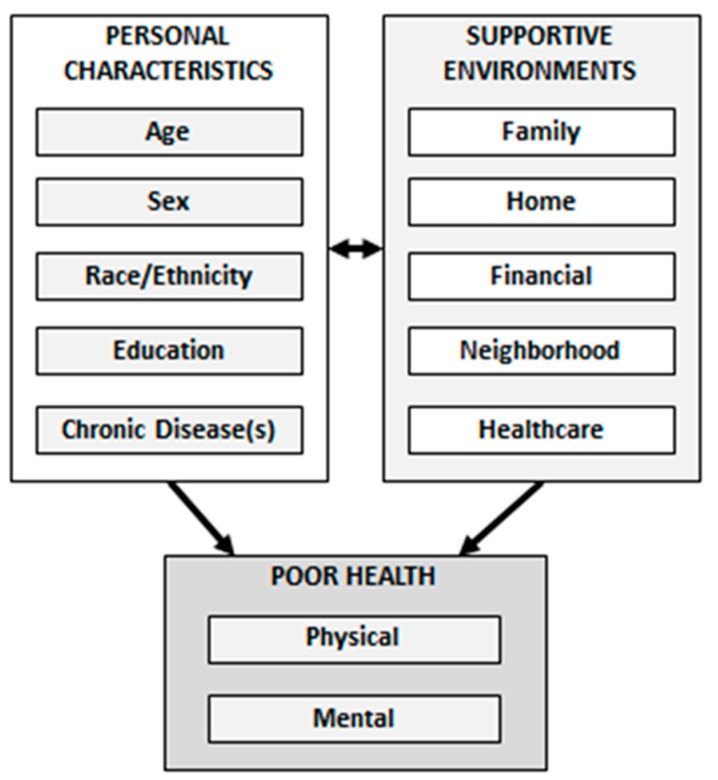
Conceptual Framework of personal characteristics and environments associated with poor health status.

The purposes of this study were to investigate associations of personal characteristics and supportive environments on poor health among older adults aged 65 and over. More specifically, this study examines factors associated with poor physical health, poor mental health, and poor combined physical/mental health. Personal characteristics examined were age, sex, race/ethnicity, education, and number of chronic diseases. Supportive environments included were: family, home, financial, neighborhood, and healthcare. An aging population appropriate conceptual framework of personal characteristics and environments associated with poor health status was used in this study (see Figure 1). The framework postulates that personal characteristics and supportive environments have direct influences on health status. However, the model also reflects how individuals with different personal characteristics may influence, or be influenced by, their various environment types. The constructs and pathways of this framework are supported by considerable research examining social determinants of health [33,34,35,36,37,38,39,40,41,42,43,44].

## 2. Materials and Methods

### 2.1. Study Participants and Procedures

Data from the 2013 Regional Healthcare Partnership—Region 17 Health Assessment were used for this study [45]. Data were collected from adults residing in one of the following Texas counties: Brazos, Burleson, Grimes, Leon, Madison, Montgomery, Robertson, Walker and Washington. The household survey was conducted in 2013 using a 24-page survey instrument developed by 39 community stakeholders. The targeted number of participants was determined per each county, to facilitate county-specific analyses. A random sample of 36,000 households was selected to participate in the region. To recruit participants, letters were sent to the household. A week later, telephone calls were made to solicit participation. Participation within the household was randomized by using the “next birthday method” among adult residents. Following the telephone call, a packet was mailed to the identified recipient, which included the survey instrument (in English or Spanish), instructions, and a self-addressed stamped envelope. No additional incentives were provided for participation. Of the 36,000 households selected, 24,768 were reached by telephone. Of those, 12,177 households agreed to complete an instrument; however, 5230 actually returned a completed instrument [45]. 

Of the 4965 participants who reported age in the dataset, we initially omitted those younger than age 65 years based on the study purposes (*n* = 3367). Of the remaining 1598 adults aged 65 years and older, cases were omitted for missing data on variables of interest. More specifically, cases were omitted for missing data on days physical/mental health not good (*n* = 76), education (*n* = 17), marital status (*n* = 4), number of people living in the household (*n* = 46), neighborhood support (*n* = 86), and healthcare access (*n* = 238). Because some cases had missing data on more than one of these variables, the final analytic sample was 1319 adults aged 65 years and older. 

Institutional Review Board approval was granted for this secondary data analyses from Texas A&M University and The University of Georgia (#00004540). 

### 2.2. Data and Measures

#### 2.2.1. Dependent Variables 

The three dependent variables used in this study were comprised of responses from two separate items from the CDC Healthy Days Scale [46]. Participants were asked, “Now thinking about your physical health, which includes physical illness and injury, for how many days during the past 30 days was your physical health not good?” Participants’ responses could range from 0 day to 30 days [46]. Then, participants were asked, “Now thinking about your mental health, which includes stress, depression, and problems with emotions, for how many days during the past 30 days was your mental health not good?” Again, participants’ responses could range from 0 day to 30 days. These continuous variables were dichotomized to indicate poor health (~50% or more of the past month). Those who reported their physical or mental health was not good for 0 to 13 days were considered to be in non-poor health. Those who reported their physical or mental health was not good for 14 to 30 days were considered to be in poor health. Then, these two continuous items (i.e., poor physical health days, poor mental health days) were summed and recoded to create a single variable ranging from 0 day to 30 days indicating the number of days the participant reported physical and/or mental health being not good. Again, this continuous variable was dichotomized (i.e., 0–13 days = non-poor health; 14–30 days = poor health). The CDC recommends that cut-points for these measures be determined by researchers based on hypothesized severity or other rationales (see http://www.cdc.gov/hrqol/faqs.htm#11), and these cut-points of 14 days have been used previously [47,48].

#### 2.2.2. Family Environment

Participants were asked to report their marital status, which was then dichotomized into the categories of married and not married. Participants were also asked to report the number of people living in their house (including themselves). This variable ranged from 1 person (self) to 9 people and was treated continuously in analyses.

#### 2.2.3. Home Environment 

To assess the conditions in the home in which participants resided, they were asked to respond to seven items. Participants were asked, “In the past 12 months has this house, apartment, or mobile home had a severe problem with any of the following: plumbing, heating/cooling, electricity (24 h without that service); mice, rats, or cockroaches; holes in floor; broken plaster or peeling paint (interior); roof (such as holes, leaks, or sagging); broken windows; mold.” All items the participant endorsed were summed to create a continuous variable (range 0 to 7 house-related problems).

#### 2.2.4. Financial Environment 

Participants were asked to report the number of financial-related services used in their household. When answering these items, participants were informed the items related to “you and all those living in your household.” Participants were asked, “In the past 12 months, each community service listed below, circle 1 if household members did not need this service, 2 if any household member needed and used this service, or 3 if any household members needed, but did not use this service.” Items were collapsed to form two categories, “did not need” and “needed.” Participants responded to items related to: Financial assistance or welfare (unemployment, TANF, social security disability-SSI); Utility assistance; Financial assistance for auto, appliance, or home repair; or weatherization; and Food, meal, and nutrition services (such as Meals-On-Wheels). All items the participant endorsed were summed to create a continuous variable (range 0 to 4 financial services needed).

#### 2.2.5. Neighborhood Environment

Based on Rural-Urban Continuum Codes (RUCC), counties in this region were coded as rural or urban [49,50]. Participants were also asked three questions to assess their perceptions about neighborhood support. Participants were asked to rate the following using a 4-point Likert-type scale (strongly agree to strongly disagree): “People in this community are willing to help their neighbors;” “this is a close-knit community;” and “People in this community can be trusted.” The internal consistency reliability coefficient (Cronbach’s alpha) for this 3-item scale was 0.834. These scores were summed to create a composite Neighborhood Support Scale, which ranged from 3 to 12, with higher scores indicating better neighborhood support.

#### 2.2.6. Healthcare Environment

Participants were asked, “Is there a specific doctor, nurse practitioner, physician assistant, or alternative health provider (chiropractor, homeopath, acupuncturist, curandero, etc.) that you consider to be your regular health care provider?” Responses choices were no and yes. Participants were also asked four questions to assess their access to healthcare. Participants were asked to rate the following using a 6-point Likert-type scale (very poor to excellent): “Your access to healthcare whenever you need it;” “Your ability to make an appointment with and see specialists if needed;” “Your access to hospital care if you need it;” and “Your access to mental health care if you need it.” The internal consistency reliability coefficient (Cronbach’s alpha) for this 4-item scale was 0.924. These scores were summed to create a composite Healthcare Access Scale, which ranged from 4 to 24, with higher scores indicating better healthcare access. 

#### 2.2.7. Personal Characteristics

Participant characteristics of interest in this study included age (range 65 to 96 years), sex (male, female), race/ethnicity (non-Hispanic white, African American, Hispanic, other/multiple), and education (less than high school, high school graduate or higher). Participants were also asked, “Has a medical care provider (physician, nurse practitioner or physician assistant) ever told you that you had any of the following health problems?” Participants were also asked to self-report their chronic conditions from a list of 16 health problems (scored no/yes). Examples of health conditions included hypertension, congestive heart failure, diabetes, cancer, asthma, arthritis, depression. All items the participant endorsed were summed to create a continuous variable (range 0 to 16 conditions). 

### 2.3. Statistical Methods

All analyses were performed using SPSS version 24 (IBM, Armonk, NY, USA). Frequencies and descriptive statistics were calculated for all variables of interest and compared based on the dependent variable categories. Chi square tests were used to assess distribution differences for categorical variables. Independent sample *t*-tests were used to assess mean differences for continuous and count variables. Then, three binary logistic regression analyses were performed. The first model examined personal characteristics and environmental factors associated with reporting poor physical health. The second model examined personal characteristics and environmental factors associated with reporting poor mental health. Then, the third model examined personal characteristics and environmental factors associated with reporting poor combined physical/mental health. For all binary regression models, participants who reported 0 to 13 days of poor health (i.e., physical, mental, physical/mental) served as the referent group. For all analyses, alpha less than 0.05 were considered to represent statistical significance.

## 3. Results

Table 1 presents sample characteristics for the current study. Over 15% of participants self-reported having 14 to 30 days of poor physical health in the past month, 7.5% reported having 14 to 30 days of poor mental health in the past month, and 20.3% reported having 14 to 30 days of poor combined physical/mental health in the past month. On average, participants were aged 70.19 (±5.13) years. A majority of participants were female (59.1%), non-Hispanic white (91.4%), and had at least a high school education (70.1%). On average, participants self-reported having 3.45 (±2.11) chronic conditions. Approximately 73% of participants were married and lived with 2.00 (±0.82) other people, on average. The majority of participants lived in a rural area (67.2%) and had a regular healthcare provider (91.3%). 

When comparing participant and environmental characteristics by poor physical health, a larger proportion of participants who had less than a high school education (χ^2^ = 21.08, *p* < 0.001), were unmarried (χ^2^ = 9.97, *p* = 0.002), resided in a rural area (χ^2^ = 4.70, *p* = 0.030), and did not have a regular healthcare provider (χ^2^ = 5.17, *p* = 0.023) reported poor physical health. On average, participants in poor physical health were older (*t* = −1.98, *p* < 0.049) and reported more chronic conditions (*t* = −8.71, *p* < 0.001). On average, participants in poor physical health reported significantly more house problems (*t* = −4.19, *p* < 0.001), more financial support needs (*t* = −5.09, *p* < 0.001), and worse neighborhood support (*t* = 3.35, *p* = 0.001). 

When comparing participant and environmental characteristics by poor mental health, a larger proportion of participants who had less than a high school education reported poor mental health (χ^2^ = 19.50, *p* < 0.001). On average, participants in poor mental health reported more chronic conditions (*t* = −7.19, *p* < 0.001), more house problems (*t* = −3.87, *p* < 0.001), more financial support needs (*t* = −3.98, *p* < 0.001), and worse neighborhood support (*t* = 3.94, *p* < 0.001).

When comparing participant and environmental characteristics by poor combined physical/mental health, a larger proportion of participants who reported a race/ethnicity other than non-Hispanic white (χ^2^ = 8.85, *p* = 0.031), had less than a high school education (χ^2^ = 25.41, *p* < 0.001), were unmarried (χ^2^ = 12.34, *p* < 0.001), and resided in a rural area (χ^2^ = 4.15, *p* = 0.042) reported poor combined physical/mental health. On average, participants in poor combined physical/mental health reported more chronic conditions (*t* = −10.42, *p* < 0.001). On average, participants in poor combined physical/mental health reported significantly more house problems (*t* = −4.99, *p* < 0.001), more financial support needs (*t* = −5.85, *p* < 0.001), worse neighborhood support (*t* = 4.96, *p* < 0.001), and lower healthcare access (*t* = 2.30, *p* = 0.022). 

Table 2 reports findings from three binary logistic regression models examining the associations of personal characteristics with poor physical, mental, and combined physical/mental health in the 30 days (i.e., reporting 0 to 13 days of poor health served as the referent group for all models). In the first model, participants who were female (OR = 0.61), married (OR = 0.64), had more than a high school education (OR = 0.61), and had a regular healthcare provider (OR = 0.49) were less likely to report poor physical health. For each additional chronic condition (OR = 1.29), house problem (OR = 1.14), and financial support need (OR = 1.39) reported, participants were significantly more likely to report poor physical health. 

In the second model, participants who had more than a high school education were less likely to report poor mental health (OR = 0.58). For each additional chronic condition (OR = 1.36) and house problem (OR = 1.19), participants were significantly more likely to report poor mental health. Conversely, for each additional unit increase in neighborhood support, participants were significantly less likely to report poor mental health (OR = 0.86).

In the third model, participants had more than a high school education were less likely to report poor combined physical/mental health (OR = 0.66). For each additional chronic condition (OR = 1.35), person living in their household (OR = 1.23), house problem (OR = 1.15), and financial support need (OR = 1.37), participants were significantly more likely to report poor combined physical/mental health. Conversely, for each additional unit increase in neighborhood support, participants were significantly less likely to report poor combined physical/mental health (OR = 0.90).

## 4. Discussion

The purpose of this study was to investigate personal characteristics and supportive environments associated with health status in older individuals aged 65 and over. Our study examined several factors explored in other research examining the role of multilevel health determinants, demonstrating the continuing relationship between these factors into later life. 

Consistent with previous research [51,52,53,54,55,56,57,58,59], this study confirms that chronic disease is an important factor influencing physical and mental health of the older individuals. According to CDC, chronic conditions are the costliest, common health issues affecting Americans, but are also preventable.

Further, as in other studies, we found that older persons with higher education were less likely to be in poor physical and mental health. There is considerable research documenting the strong association between education and health outcomes [60,61,62,63,64]. Having a higher education attainment is associated with an increased health-related knowledge, healthier lifestyle and environments, and a sense of personal control over circumstances [10]. A study with a large sample size from the National Health Measurement Study found that persons aged 35 to 89 years with lower education level had worse health-related quality of life than those with higher education level [19].This study showed that participants who were female were less likely to report poor physical health. The literature on gender differences and physical health shows conflictive results [14,15,16,65,66,67,68]. Possible explanations for the discrepancy may include differences in the measurement of physical health as well as variations in the sex-ratio and disability-related conditions among samples. Our study did not investigate gender differences in prevalence of chronic diseases; thus findings discrepancies warrant further examination.

This study is unique because it examines the associations of 5 different types of environments on physical and mental health. All environments (family, home, financial, neighborhood, and healthcare environments) were significantly associated with poorer health in our study population. Consistent with the literature [69,70,71], results in this study indicate that those who were married were less likely to report poorer physical health than those not married. 

In our study participants that had a regular healthcare provider were less likely to report poorer physical health than those experiencing opposite circumstances. Other authors found a negative association between health and healthcare utilization [72,73,74,75,76,77]. These discrepancies may be related to health-status measurements and disability-related conditions among samples. Our study found that higher financial needs were significantly associated with poorer physical health, which is consistent with previous research [17,78,79,80]. An intriguing finding in our study was that for each additional person living at home, participants were significantly more likely to be in poorer physical and mental health. There are two possible explanations for this result. One explanation is that the elders in the household are carrying the burden of financially supporting others in the family. Research conducted in California indicates that when an older adult supports one adult child at home, their cost of living increases by at least 50% [81]. A second explanation is that because of their poor health older persons tend to live with family members, besides the spouse, as a safety net strategy. For instance, The Chinese Longitudinal Healthy Longevity Survey found that for older individuals living with family members was positive for their self-rated health, but it was associated also with a higher risk of functional and cognitive impairment [82]. Other studies found an association between functional impairment and not living alone at home [83]. 

Our findings indicate that participants perceiving to live in a neighborhood with trusted and helpful neighbors were less likely to report poor mental health. This is consistent with previous research [84,85,86,87,88,89,90]. A study conducted using data from the 2010 National Outcome and Assessment Information Set found that living alone and insufficient social support were predictors of hospitalizations and poor health among elderly individuals [53]. Research in Australian rural communities discovered that increased community support and personal support were predictors of well-being [23].

Study findings related to housing conditions and physical and mental health resonate with previous research [40,41,42,43]. One study conducted with adults determined a positive impact between housing improvements and physical and mental health [90].

Given, our findings surrounding social ecological factors [91] and structural and social determinants of health [44] being significantly associated with poor physical and mental health, we suggest further research in this area. For example, previous work in Texas has looked at health behaviors predictive of poor health, namely physical activity as it relates to older adults [92,93]. These findings show the importance of both perceived and objectively measured characteristics of one’s environment, especially as environments perceived as more cohesive or measured objectively as walkable [92] have been shown to be associated with a greater likelihood of meeting certain thresholds of physical activity through walking. Walking is particularly important for older adults, as it has been shown to be the most common form of physical activity among this age group [94].

### Limitations

This study was based on a cross-sectional design; therefore, causal inferences cannot be made. Future research should incorporate longitudinal data to determine causal associations throughout the life course. Although one of the strengths of this study is the use of a large data set, due to the sampling technique used and because data were collected in only one region in Texas, findings of this study may be generalized only to older adults living in areas with similar demographic characteristics to the study participants. It should be noted that there was considerable case attrition when applying inclusion criteria for this study (approximately 400 cases were omitted because of missing data). About half of cases omitted were missing data related to their access to healthcare, which may have biased our sample towards those with some form of health insurance (lack of insurance is typically associated with those of less education, less affluence, and certain races/ethnicities). Further, given the vast majority of older adults in the target area reported being white, these results are limited in generalization to other more diverse racial or ethnic groups. Future research should incorporate a more diverse group of individuals for greater generalizability. Although health status was measured using a valid, reliable instrument, dependent and independent variables were measured using a self-report instrument, which could have led to some biases. For instance, data was not collected to determine if study participants were actually receiving social services they reported needing. In addition, not all variables were asked that would give context about each environment (independent variables) and its contribution to the health status of participants. Measures of family and neighborhood environments did not elicit additional information from participants which could have been useful to investigate more in depth the association between these constructs and health status. Finally, other potential protective environments relevant to health status of older individuals, such as the built-environment and social network, were not included in this study.

## 5. Conclusions

Despite its limitations, this study shows that multidimensional factors including personal characteristics and protective environments, such as family, home, finances, neighborhood, and access to healthcare, all come into play to determine poor or good physical and mental health of older individuals. Given the growth in older adults [1], this study is especially timely for policy makers and other stakeholders seeking to identify risk factors for poor health among older adults. This study provides relevant information that may inform public health interventions aiming to improve physical and mental health among older adults. Our study findings suggests a need for chronic disease-self management interventions tailored for older adults, which have been shown to be effective in reducing and controlling chronic conditions [95,96]. Families could also be exposed to programs educating and empowering them to become a strong, positive support for their older adult co-habitant. Community and public health efforts could focus activities and events at local venues to promote neighborhood cohesion, capacity building, and social support for older persons. Finally, access to healthcare could be increased by providing mobile clinics or telehealth services, as well as providing elders with low-cost transportation to attend physician visits.

## Figures and Tables

**Table 1:** Personal and Environment-Related Characteristics by Health Days Not Good.

		Physical Days Health Not Good				Mental Days Health Not Good				Combined Days Health Not Good			
	Total (*n* = 1319)	0 to 13 Days (*n* = 1117)	14 to 30 Days (*n* = 202)	χ^2^ or *t*	*p*	0 to 13 Days (*n* = 1220)	14 to 30 Days (*n* = 99)	χ^2^ or *t*	*p*	0 to 13 Days (*n* = 1051)	14 to 30 Days (*n* = 268)	χ^2^ or *t*	*p*
**PERSONAL CHARACTERISTICS**													
Age	70.19 (±5.31)	70.06 (±5.23)	70.91 (±5.72)	−1.98	**0.049**	70.18 (±5.29)	70.30 (±5.60)	−0.23	0.822	70.06 (±5.21)	70.68 (±5.69)	−1.61	0.108
Sex: Male	40.9%	40.3%	44.6%	1.29	0.256	41.6%	33.3%	2.56	0.110	41.6%	38.4%	0.87	0.350
Sex: Female	59.1%	59.7%	55.4%			58.4%	66.7%			58.4%	61.6%		
Race/Ethnicity: Non-Hispanic White	91.4%	92.2%	87.1%	6.21	0.102	92.0%	84.8%	6.86	0.077	92.6%	86.9%	8.85	**0.031**
Race/Ethnicity: African American	2.3%	2.0%	4.0%			2.0%	5.1%			1.9%	3.7%		
Race/Ethnicity: Hispanic	1.6%	1.5%	2.0%			1.6%	2.0%			1.4%	2.2%		
Race/Ethnicity: Other/Multiple	4.7%	4.3%	6.9%			4.4%	8.1%			4.1%	7.1%		
Education: Less than High School	29.9%	27.5%	43.6%	21.08	**<0.001**	28.4%	49.5%	19.50	**<0.001**	26.7%	42.5%	25.41	**<0.001**
Education: High School or More	70.1%	72.5%	56.4%			71.6%	50.5%			73.3%	57.5%		
Number of Chronic Conditions	3.45 (±2.11)	3.24 (±2.03)	4.60 (±2.19)	−8.71	**<0.001**	3.30 (±2.00)	5.21 (±2.58)	−7.19	**<0.001**	3.13 (±1.94)	4.70 (±2.27)	−10.42	**<0.001**
**FAMILY ENVIRONMENT**													
Marital Status: Not Married	26.6%	25.0%	35.6%	9.97	**0.002**	26.1%	33.3%	2.48	0.116	24.5%	35.1%	12.34	**<0.001**
Marital Status: Married	73.4%	75.0%	64.4%			73.9%	66.7%			75.5%	64.9%		
Number of People Living in Household	2.00 (±0.82)	1.98 (±0.75)	2.11 (±1.12)	−1.63	0.105	1.99 (±0.78)	2.19 (±1.17)	−1.73	0.086	1.97 (±0.74)	2.11 (±1.07)	−1.95	0.052
**HOME ENVIRONMENT**													
Number of House Problems	0.51 (±1.13)	0.43 (±0.98)	0.94 (±1.66)	−4.19	**<0.001**	0.46 (±1.05)	1.13 (±1.71)	−3.87	**<0.001**	0.41 (±0.96)	0.90 (±1.55)	−4.99	**<0.001**
**FINANCIAL ENVIRONMENT**													
Number of Financial Support Needs	0.23 (±0.68)	0.17 (±0.56)	0.56 (±1.08)	−5.09	**<0.001**	0.20 (±0.63)	0.63 (±1.06)	−3.98	**<0.001**	0.15 (±0.54)	0.53 (±1.02)	−5.85	**<0.001**
**NEIGHBORHOOD ENVIRONMENT**													
Lives in Rural Area: No	32.8%	34.0%	26.2%	4.70	**0.030**	33.4%	26.3%	2.09	0.148	34.2%	27.6%	4.15	**0.042**
Lives in Rural Area: Yes	67.2%	66.0%	73.8%			66.6%	73.7%			65.8%	72.4%		
Neighborhood Support Scale	9.15 (±1.59)	9.22 (±1.54)	8.77 (±1.79)	3.35	**0.001**	9.21 (±1.54)	8.42 (±1.94)	3.94	**<0.001**	9.27 (±1.53)	8.69 (±1.74)	4.96	**<0.001**
**HEALTHCARE ENVIRONMENT**													
Has Regular Healthcare Provider: No	8.7%	8.0%	12.9%	5.17	**0.023**	8.8%	8.1%	0.06	0.815	8.1%	11.2%	2.59	0.108
Has Regular Healthcare Provider: Yes	91.3%	92.0%	87.1%			91.2%	91.9%			91.9%	88.8%		
Healthcare Access Scale	20.57 (±3.76)	20.65 (±3.61)	20.13 (±4.49)	1.55	0.124	20.63 (±3.66)	19.90 (±4.75)	1.49	0.140	20.70 (±3.60)	20.05 (±4.30)	2.30	**0.022**

Bold text indicates significant *p*-values.

**Table 2:** Logistic regression analyses.

	Physical Days Not Good						Mental Days Not Good						Combined Physical/Mental Days Not Good					
					95% CI						95% CI						95% CI	
	Beta	S.E.	OR	*p*	Lower	Upper	Beta	S.E.	OR	*p*	Lower	Upper	Beta	S.E.	OR	*p*	Lower	Upper
Age	0.02	0.02	1.02	0.218	0.99	1.05	−0.01	0.02	0.99	0.729	0.95	1.04	0.01	0.01	1.01	0.375	0.99	1.04
Sex: Male	--	--	1.00	--	--	--	--	--	1.00	--	--	--	--	--	1.00	--	--	--
Sex: Female	−0.50	0.18	0.61	**0.004**	0.43	0.85	0.16	0.25	1.17	0.527	0.72	1.89	−0.11	0.16	0.90	0.492	0.65	1.23
Race/Ethnicity: Non-Hispanic White	--	--	1.00	--	--	--	--	--	1.00	--	--	--	--	--	1.00	--	--	--
Race/Ethnicity: African American	−0.21	0.34	0.81	0.536	0.41	1.59	−0.53	0.43	0.59	0.218	0.26	1.37	−0.28	0.32	0.75	0.371	0.41	1.40
Race/Ethnicity: Hispanic	−0.19	0.56	0.82	0.731	0.27	2.49	−0.29	0.68	0.75	0.672	0.20	2.82	−0.40	0.53	0.67	0.453	0.24	1.90
Race/Ethnicity: Other/Multiple	−0.09	0.68	0.92	0.897	0.24	3.45	−0.27	0.88	0.77	0.763	0.14	4.33	−0.01	0.61	0.99	0.981	0.30	3.23
Education: Less than High School	--	--	1.00	--	--	--	--	--	1.00	--	--	--	--	--	1.00	--	--	--
Education: High School or More	−0.49	0.18	0.61	**0.006**	0.43	0.87	−0.54	0.23	0.58	**0.021**	0.37	0.92	−0.41	0.16	0.66	**0.012**	0.48	0.91
Number of Chronic Conditions	0.25	0.04	1.29	**<0.001**	1.19	1.39	0.31	0.05	1.36	**<0.001**	1.24	1.50	0.30	0.04	1.35	**<0.001**	1.26	1.45
Marital Status: Not Married	--	--	1.00	--	--	--	--	--	1.00	--	--	--	--	--	1.00	--	--	--
Marital Status: Married	−0.44	0.21	0.64	**0.034**	0.43	0.97	−0.11	0.29	0.90	0.702	0.51	1.57	−0.38	0.19	0.69	0.050	0.47	1.00
Number of People Living in Household	0.18	0.09	1.20	0.055	1.00	1.44	0.20	0.12	1.22	0.093	0.97	1.54	0.21	0.09	1.23	**0.021**	1.03	1.46
Number of House Problems	0.13	0.06	1.14	**0.036**	1.01	1.29	0.18	0.08	1.19	**0.025**	1.02	1.39	0.14	0.06	1.15	**0.020**	1.02	1.30
Number of Financial Support Needs	0.33	0.11	1.39	**0.002**	1.13	1.71	0.17	0.13	1.18	0.204	0.91	1.53	0.31	0.10	1.37	**0.002**	1.12	1.68
Lives in Rural Area: No	--	--	1.00	--	--	--	--	--	1.00	--	--	--	--	--	1.00	--	--	--
Lives in Rural Area: Yes	0.24	0.19	1.27	0.206	0.88	1.83	0.15	0.26	1.17	0.549	0.71	1.93	0.16	0.17	1.18	0.336	0.85	1.64
Neighborhood Support Scale	−0.05	0.05	0.95	0.362	0.86	1.06	−0.16	0.07	0.86	**0.026**	0.74	0.98	-0.11	0.05	0.90	**0.029**	0.82	0.99
Has Regular Healthcare Provider: No	--	--	1.00	--	--	--	--	--	1.00	--	--	--	--	--	1.00	--	--	--
Has Regular Healthcare Provider: Yes	−0.72	0.27	0.49	**0.007**	0.29	0.82	0.07	0.42	1.07	0.876	0.47	2.42	−0.46	0.26	0.63	0.072	0.38	1.04
Healthcare Access Scale	0.03	0.02	1.03	0.179	0.99	1.08	0.02	0.03	1.02	0.502	0.96	1.08	0.02	0.02	1.02	0.331	0.98	1.06
	Nagelkerke R Square = 0.167						Nagelkerke R Square = 0.181						Nagelkerke R Square = 0.202					

Referent group: 0 to 13 days not good. Bold text indicates significant *p*-values.
